# Co-occurrence of Adult ADHD Symptoms and Problematic Internet Use and Its Links With Impulsivity, Emotion Regulation, Anxiety, and Depression

**DOI:** 10.3389/fpsyt.2022.792206

**Published:** 2022-04-13

**Authors:** Sarah El Archi, Servane Barrault, Paul Brunault, Aurélien Ribadier, Isabelle Varescon

**Affiliations:** ^1^University of Tours, Laboratory QualiPsy, EE1901, Tours, France; ^2^CHRU of Tours, Centre de Soins d'Accompagnement et de Prévention en Addictologie (CSAPA 37), Tours, France; ^3^Université Paris Cité, Laboratory of Psychopathology and Health Processes, Boulogne-Billancourt, France; ^4^CHRU of Tours, Service d'Addictologie Universitaire, Équipe de Liaison et de Soins en Addictologie, Tours, France; ^5^University of Tours, UMR 1253, iBrain, INSERM, Tours, France

**Keywords:** Internet Addiction, ADHD, impulsivity, anxiety disorders, depressive disorders, dual diagnosis

## Abstract

The co-occurrence of attention-deficit/hyperactivity disorder (ADHD) and problematic Internet use (PIU) is associated with increased severity of PIU and poorer treatment outcomes. The main objective of this study was to examine the association between PIU and adult ADHD symptoms and determine whether adult ADHD symptoms were a predictor of PIU in the general adult population. We also examined the potential mediating role of the dimensional psychopathological factors, including anxiety, depression, impulsivity, and emotion regulation, in this relationship. To achieve these aims, we recruited 532 regular Internet users online from the general adult population. The participants completed an online questionnaire assessing PIU (Internet Addiction Test), anxiety and depression symptoms (Hospital Anxiety and Depression Scale), adult ADHD symptoms (Adult ADHD Self-Report Scale-V1.1), emotion regulation (Emotion Regulation Questionnaire), and impulsivity (UPPS-P Impulsive Behavior Scale). We conducted a multiple regression analysis to determine the predictors of PIU and mediation analyses to identify the psychopathological mediators of the association between adult ADHD symptoms and PIU. PIU was observed in 17.9% of our sample. A significantly higher proportion of respondents with PIU screened positive for adult ADHD symptoms compared to respondents without PIU (50.5 vs. 21.7%; *p* < 0.001). Individuals with PIU reported significantly higher scores than those without PIU for anxiety and depressive symptoms, impulsivity, and the emotion regulation strategy of expressive suppression. Additionally, they had significantly lower scores than those without PIU on cognitive reappraisal than non-problematic Internet users. In addition to adult ADHD symptoms, the multiple regression analysis revealed that PIU was also positively predicted by depressive symptoms, positive urgency, lack of perseverance, and expressive suppression, and is negatively predicted by cognitive reappraisal and negative urgency. The mediation analysis showed that lack of perseverance, positive urgency, and depressive and anxiety symptoms were partial mediators of the relationship between adult ADHD symptoms and PIU. Our results highlight the significant co-occurrence of PIU and adult ADHD symptoms. This study also provides support for a theoretical model in which impulsivity dimensions, emotion regulation strategies, as well as the tendency to anxiety and depressive symptoms, may play a mediating role in this co-occurrence. In summary, the findings emphasize the need to assess these psychological characteristics in problematic Internet users, as they can be a factor of clinical complexity, as well as the importance of targeting them as part of integrated interventions for both adult ADHD symptoms and PIU.

## Introduction

Problematic Internet use (PIU) is a highly prevalent problematic behavior, especially among young people. It was first described by Young (1, 2), who defined it as an impulse-control disorder that does not involve an intoxicant. According to Spada (3), the two main features of PIU are (1) preoccupation with a loss of control over Internet use, and (2) negative consequences. A meta-analysis based on 133 surveys across 31 countries conducted between 2003 and 2018 reported PIU prevalence rates ranging from 0.5 to 40.0%, with a pooled prevalence of 8.9% in eastern countries and 4.6% in western countries (4). As with other problematic behaviors (such as sexual or food addictions), PIU is not recognized as an addictive disorder by international diagnostic classifications [Diagnostic and Statistical Manual of Mental Disorders 5th edition, DSM-5 (5); International classification of diseases 11th revision, ICD-11 (6)]. Therefore, in order to further our understanding of PIU, it may be beneficial to draw inspiration from other problematic behaviors that are included in international classifications, such as problem gambling. We thus based the rationale for our study on the seminal work of Blaszczynski and Nower (7), which proposed a three-pathway model of problem gambling. Firstly, this model includes a behaviorally conditioned pathway, referring to the effects of conditioning, distorted cognitions and poor decision-making. Secondly, the emotionally vulnerable pathway refers to individuals with premorbid anxiety or depression, and a history of poor coping skills. Finally, the antisocial-impulsivity pathway includes individuals with characteristics of impulsivity, antisocial personality disorder, and attention deficit.

In terms of the comorbidities associated with PIU, previous investigations have yielded divergent results for some PIU comorbidities. However, a meta-analysis conducted in 2014 reported a low level of between-study heterogeneity regarding the comorbidity of ADHD and PIU (8). ADHD is a neurodevelopmental disorder characterized by inattention and hyperactivity-impulsivity (5). ADHD affect 5.0–7.0% of children (9, 10) before the age of 12 and persist in adulthood in ~65% of cases (11). Other ADHD symptoms include high reward sensitivity, high sensation seeking, impaired cognitive control, and urgency, which may also be involved in the onset or maintenance of problematic behaviors. Specifically, Yoo et al. (12) found a significant link between PIU and ADHD in children and showed that ADHD was an important risk factor for PIU. Similar results have also been found with adults (13). Furthermore, results of a meta-analysis conducted in 2017 indicated that individuals with PIU are two and a half times more likely to be diagnosed with ADHD (prevalence ranging from 19.5 to 42.5%) compared with individuals without PIU (prevalence ranging from 4.6 to 15.2%) (14). Finally, both inattention and hyperactivity-impulsivity are more severe in individuals with PIU than healthy controls (14). Taken together, these results support the hypothesis of a positive association between PIU and ADHD.

The psychopathological mechanisms underlying the co-occurrence of problematic behaviors and ADHD are still unclear. However, identifying the psychological characteristics involved in this comorbidity may be useful for developing targeted interventions to improve treatment outcomes and prevent problematic behaviors in individuals with ADHD. Based on the three-pathway model proposed by Blaszczynski and Nower (7), we hypothesized that certain psychopathological factors lead ADHD individuals to engage in problematic behaviors, such as PIU. For example, the antisocial-impulsivity and emotionally vulnerable pathways show shared psychological factors between ADHD and problematic behaviors. Therefore, impulsivity, the use of maladaptive emotion regulation strategies, and anxiety and depressive symptoms could be interesting candidates as mediators of the association between ADHD and PIU.

In terms of anxiety and depression symptoms, a short-term longitudinal study found that anxiety and depressive symptoms positively predicted PIU in 12- to 18-year-old adolescents (15). According to LaRose et al. (16), Internet use may be a way for individuals with low levels of stimulation, such as those with depressive disorders, to alleviate their dysphoria. Therefore, depression associated with impaired self-regulation may lead to difficulties with controlling Internet use, thus causing PIU. Emotion regulation refers to “the processes by which individuals influence which emotions they have, when they have them, and how they experience and express these emotions” (17). Emotion dysregulation is prevalent in individuals with ADHD (18), and these difficulties with emotion regulation may lead to the use of maladaptive strategies, such as emotion suppression, and ultimately to PIU. Similarly, previous studies have suggested that emotion dysregulation may contribute to problematic behaviors, such as addictive disorders (19–22).

Impulsive actions may provide immediate rewards and alleviate negative emotions (23), which are a significant feature of ADHD. Moreover, previous studies have suggested that the association between ADHD and problematic behaviors may be mediated by impulsivity (24) or anxiety and depressive symptoms (25). Taken together, the previous research is in line with the hypotheses that negative affectivity (i.e., anxiety and depressive symptoms), the use of maladaptive emotion regulation strategies, and impulsivity are psychological features that may partially explain the association between ADHD and PIU. However, there is a lack of studies investigating these hypotheses together in the specific population of individuals with PIU.

In this study, we aimed to investigate the prevalence of the co-occurrence of PIU and adult ADHD symptoms and the independent and mediation effects of psychological factors on the relationship between these two conditions, especially in terms of negative affectivity (anxiety and depressive symptoms), emotion regulation, and impulsivity. We hypothesized that respondents with PIU may have a higher level of impulsivity (especially in terms of urgency) and negative affectivity (anxiety and depressive symptoms) and may tend to use maladaptive emotion regulation strategies. We expected that these dimensional variables predict PIU severity, and may mediate this association between adult ADHD symptoms and PIU severity.

## Materials and Methods

The research was conducted in accordance with the Helsinki Declaration, as revised in 1989. Prior to inclusion in the study, all participants provided written informed consent once the procedure had been fully explained to them. The protocol was approved by the Institutional Review Board (France) in April 2019 (IRB number: 2019-03-01).

### Population and Procedure

This cross-sectional study was conducted online. The participants were recruited over ~1 year via the social media of three psychology students and two researchers (i.e., Facebook, Twitter, blogs, and forums) of the University of Tours (France). The participants were self-selected, and their participation was voluntary. They were considered eligible for inclusion if they were at least 18 years old, used the Internet at least once a week, gave their informed and signed consent, and completed the questionnaire in its entirety.

The participants were provided with a brief text giving them information about the study, including the aims and methods, the inclusion criteria (as defined above), and the confidential and anonymized nature of the data. The eligible participants were assessed using self-administered questionnaires, which were designed and completed online using LimeSurvey software. In total, 544 participants completed the questionnaire. Twelve participants were excluded overall because of being aged under 18 years old (*N* = 1) and having missing data (*N* = 11). Therefore, our final sample comprised 532 Internet users.

### Measures

#### Socio-Demographic and Internet Activity Data

We collected socio-demographic data, including age, gender, marital status, and employment status. The participants were asked to report their marital status (among the proposals specified in Table 1) and their employment status: “employed” (including full-time employment, part-time employment and irregular work), “unemployed” (including unemployed and retired), “students” and “other situations” (included disabled and others situations). The participants were also asked to report their favorite Internet activity: e-mail-related activity, social media use (i.e., Facebook, Twitter, Instagram), taking or looking at photographs, watching videos, playing games, using search engines, reading news, downloading, online purchasing, watching pornography online, online gambling, and using online dating sites. There were two questions regarding Internet activities. The first one was: “Was are the activities you practice online?” and the second: “Please report your three favorites online activities (the ones you spend the most time on, or your favorite if you spend the same amount of time on several activities), and classify them from 1 (first favorite) to 3 (third favorite).” For each question, participants had the choice in the list of activities mentioned above.

**Table 1:** Socio-demographic data and independent variables: comparison of PIU and non-PIU individuals.

	**PIU** **(*N* = 95)**	**Non-PIU** **(*N* = 437)**	**Statistics**	
	**[% or mean (SD)]**	**[% or mean (SD)]**	**(χ^2^ or U)**	***p***
Gender (% women)	66.0	74.8	1.159	0.282
Age	26.9 (11.2)	27.3 (10.0)	18695.5	0.128
Marital status			10.159	0.006^*^
Married/partnered	31.6	48.1		
Single	62.1	49.2		
Divorced/separated	6.3	2.7		
Occupation			21.205	<0.001^*^
Employed	24.2	42.6		
Unemployed	6.3	4.3		
Students	60.0	51.0		
Other situations	9.5	2.1		
Problematic internet use (IAT)	57.8 (6.6)	34.8 (7.8)	41515.0	<0.001^*^
Anxiety and depression (HADS total)	12.4 (5.0)	9.5 (4.8)	13438.5	<0.001^*^
Anxiety symptoms	7.7 (3.2)	6.5 (3.2)	15755.0	<0.001^*^
Depression symptoms	4.6 (2.8)	3.1 (2.5)	13475.5	<0.001^*^
Impulsivity (UPPS-P total)	48.9 (7.8)	45.0 (7.5)	14436.5	<0.001^*^
Negative urgency	10.9 (2.9)	10.5 (3.1)	19476.0	0.343
Positive urgency	10.8 (2.6)	10.1 (2.7)	17546.0	0.017^*^
Lack of premeditation	8.2 (2.4)	7.3 (2.3)	15637.5	<0.001^*^
Sensation seeking	10.2 (3.0)	10.3 (2.9)	20323.0	0.748
Lack of perseverance	8.7 (3.0)	6.8 (2.4)	12936.5	<0.001^*^
Adult ADHD symptoms (ASRS)	3.4 (1.5)	2.4 (1.4)	12662.0	<0.001^*^
Expressive suppression (ERQ)	17.4 (5.5)	14.9 (5.2)	14423.0	<0.001^*^
Cognitive reappraisal (ERQ)	23.6 (9.0)	27.6 (6.9)	15120.0	<0.001^*^

* *p ≤ 0.05*;

*χ^2^ = chi-squared test; U, Mann–Whitney coefficient; PIU, problematic internet use assessed by the Internet Addiction Test; IAT, Internet Addiction Test; HADS, Hospital Anxiety and Depression Scale; ASRS, Adult ADHD Self-report Scale; ERQ, Emotion Regulation Questionnaire*.

#### Problematic Internet Use

We assessed PIU using the Internet Addiction Test [IAT; (2), French version by (26)], which is a 20-item self-report scale for identifying individuals who exhibit addictive-like behavior in their Internet use. This scale is based on the Diagnostic and Statistical Manual of Mental Disorders Text Revision Fourth Edition [DSM-IV-TR; (27)] criteria for pathological gambling: loss of control over Internet use, significant impact of Internet use in different areas of life, and tolerance and dependence symptoms. Each item is rated on a 5-point Likert scale, ranging from “rarely” to “always,” and the total score is obtained by summing the scores of all the items. A score over 50 suggests that the individual experiences problems with Internet usage (26). In this study, we used a cut-off score of 50 to differentiate between individuals with and without self-reported PIU. The IAT has excellent internal consistency (α = 0.86, in the current study) and is the most widely used self-administered questionnaire for evaluating PIU.

#### Adult ADHD Symptoms

Adult ADHD symptoms was screened using the self-reported Adult ADHD Self-Report Scale-V1.1 (ASRS-V1.1), which is a 6-item self-administered questionnaire designed with the support of the World Health Organization to screen for adult ADHD symptoms in both community surveys and clinical settings based on criteria of the DSM-IV-TR (28). The items are rated on a 5-point Likert scale, with a cut-off score for each item. The ASRS is an effective tool for screening adults for ADHD symptoms, with a Cronbach's α ranging from 0.63 to 0.72 in the overall population (29) and good internal consistency (α = 0.84) and construct validity in adult patients with addictive disorders (30). The current study internal consistency was 0.69. The presence of at least four significant items (i.e., above the defined cut-off scores) suggests a high risk of adult ADHD symptoms (28). Therefore, we used this criterion to differentiate between participants with and without adult ADHD symptoms.

#### Anxiety and Depressive Symptoms

To assess anxiety and depressive symptoms, we used the Hospital Anxiety and Depression Scale [HADS; (31); French version by (32)]; this is a 14-item self-report scale that screens for both anxiety (7 items) and depression (7 items). It has good psychometric properties (31, 33), is quick to administer, and is, thus, suitable for field research. Scores of 0–7 indicate no disorder, 8–10 indicate doubtful cases, and 11 and over indicate definite cases (31). In this study, we used a cut-off score of 8 [possible disorder; (33)]. The HADS has been widely used in research and has good psychometric qualities (34). The current study internal consistency was 0.69 for anxiety and 0.47 for depression.

#### Emotion Regulation

We used the Emotion Regulation Questionnaire (ERQ) to assess emotion regulation [(35); French version by (36)]. This 10-item scale is a self-report measure of two distinct emotion regulation strategies: cognitive reappraisal (CR; transforming the way a situation is perceived in order to change its meaning and emotional impact) and expressive suppression (ES; inhibiting or reducing facial expression of emotions). Both the original version and the French version have good psychometric properties (35, 36), indicating that the ERQ is a reliable tool for assessing these strategies. Factorial and confirmatory analyses revealed a two-factor structure of the scale: 6 items assess cognitive reappraisal, and 4 items assess expressive suppression. The current study internal consistency was 0.76 for both CR and ES.

#### Impulsivity

Impulsivity was assessed using the UPPS Impulsive Behavior Scale, short version (UPPS-P) [(37), French version by (38)]. This is a 20-item self-administered questionnaire based on the UPPS model (37, 39), with one additional measure of positive urgency (23). The scale assesses five facets of impulsivity: negative urgency, positive urgency, lack of premeditation, lack of perseverance, and sensation seeking (40). The UPPS-P provides a sub-score for each facet, and higher scores indicate higher intensity of impulsivity. In the current study, the UPPS-P showed acceptable to good psychometric properties, as the Cronbach's α values were 0.83 for negative urgency, 0.77 for positive urgency, 0.78 for lack of premeditation, 0.84 for lack of perseverance, and 0.66 for sensation seeking.

### Statistical Analyses

Analyses were conducted using SPSS® version 22 (IBM Corp. Released 2013. IBM SPSS Statistics for Windows, Version 22.0, IBM Corporation, Armonk, NY, USA). Analyses were two-tailed and *p*-values ≤0.05 were considered statistically significant. Descriptive statistics were presented using percentages for ordinal variables and the means and standard deviations for continuous variables. Percentage values were analyzed using the Chi-Square test, and quantitative data (scale scores) were analyzed using the Mann–Whitney *U* test [the threshold of significant was adjusted for multiple comparisons (α' = α/number of subdimensions of the scale)].

We conducted a multiple regression analysis to determine whether the quantitative variables (adult ADHD symptoms, anxiety, depression, five impulsivity sub-dimensions, and two emotion regulation sub-dimensions) were predictors of PIU (IAT score as the dimension). As there were no latent variables in the proposed models, mediation analyses with a regression-based approach were performed using the PROCESS macro (version 3.5.3) for IMB SPSS Statistics 22 (41) rather than structural equation modeling. The regression assumptions were confirmed, outliers were removed, and normal distribution and homoscedasticity were ensured through the square root transformation of the dependent variables. Bootstrap sampling was conducted using 5,000 resamples. We assessed collinearity between variables by making sure that variance inflation factor (VIF) was under 5 as recommended (42).

The following procedure was utilized to assess the mediation effects of anxiety and depressive symptoms, emotion regulation, and impulsivity in the association between self- adult ADHD symptoms and PIU. Gender and age were adopted as covariables. In the mediation model of the effect of X on Y through M, X was adult ADHD symptoms (ASRS score), Y was PIU (IAT score as a dimension), and M was the mediator variable. We conducted 3 multiple mediations to examine the independent mediation effects of the two HADS scores, the two ERQ scores, and the five UPPS-P scores (M variables) in the association between adult ADHD symptoms (ASRS score) and PIU (IAT score). Unstandardized regression coefficients were identified: *path a* (the effect of adult ADHD symptoms on M), *path b* (the effect of M on PIU), *path c* (the total effect of adult ADHD symptoms on PIU), and *path c'* (the direct effect of adult ADHD symptoms on PIU). Overall, the indirect effect of adult ADHD symptoms on PIU was the product of *path a* and *path b*.

## Results

### Socio-Demographic Data, PIU, and Internet Activities

In our sample, the proportion of respondents with PIU was 17.9% (*N* = 95). The mean age of participants was 27.23 (SD = 10.18), and 73.9% of the sample were women. No differences in respondent with PIU were identified for age and gender (age: *U* = 18695.5; *p* = 0.13; gender: χ^2^ = 1.159*; p* = 0.28). However, there were significant differences between individuals with and without PIU in terms of their marital status and occupation, as individuals with PIU were more likely to be single (details are presented in Table 1). There were no differences (χ^2^ = 19.749; *p* = 0.14) in preferred online activity between the PIU and non-PIU groups. Overall, the most prevalent activities included the use of social media (42.9%), e-mails (20.1%), information searches (12.6%), and gaming (12.2%).

### Prevalence of Adult ADHD Symptoms

The proportion of respondents who screened positive for adult ADHD symptoms in individuals with PIU (50.5%, *N* = 48) was significantly higher than in individuals without PIU (21.7%, *N* = 95; χ^2^ = 32.9, *p* < 0.001).

### Comparison of Internet Users With and Without PIU

Table 1 presents the variable scale scores for both PIU and non-PIU individuals. Those with PIU scored significantly higher than those without PIU on every variable, except cognitive reappraisal, which was significantly higher among individuals without PIU. Only the impulsivity sub-dimensions of lack of perseverance and lack of premeditation were significantly higher for individuals with PIU than those without PIU.

### Multiple Regression Model

The multiple regression model explained 20.0% of the variance of IAT scores [*F*_(10,531)_ = 14.55; *R*^2^ = 0.22; *AdjustedR*^2^ = 0.20; *p* < 0.001]. As shown in Table 2, IAT scores were significantly predicted by HAD-depression symptoms, UPPS-positive urgency, UPPS-lack of perseverance, ASRS, and ERQ-expressive suppression scores. Additionally, UPPS-negative urgency and ERQ-cognitive reappraisal scores were a negative predictor of IAT. Details are presented in Table 2.

**Table 2:** Multiple regression model explaining IAT scores.

	**β**	**Err-type**	**b**	**Err-type**	***t*_(530)_**	***p***
OrdOrig.			5.34	0.28	19.09	<0.001^*^
Anxiety symptoms	0.07	0.04	0.02	0.01	1.56	0.12
Depression symptoms	0.12	0.05	0.04	0.02	2.62	0.009^*^
Negative urgency	−0.09	0.04	−0.03	0.01	−1.96	0.05^*^
Positive urgency	0.17	0.04	0.06	0.02	3.85	<0.001^*^
Lack of premeditation	−0.043	0.04	−0.02	0.02	−0.96	0.34
Lack of perseverance	0.11	0.05	0.04	0.02	2.27	0.02^*^
Sensation seeking	−0.05	0.04	−0.02	0.01	−1.31	0.19
Adult ADHD symptoms	0.23	0.04	0.15	0.03	5.15	<0.001^*^
Cognitive reappraisal	−0.13	0.04	−0.02	0.01	−3.22	0.001^*^
Expressive suppression	0.12	0.04	0.02	0.01	3.01	0.003^*^

* *p ≤ 0.05*;

*β, standardized coefficient; b, unstandardized coefficient; IAT, Internet Addiction Test*.

The multiple regression model conducted with the stepwise method explained 20.0% of the variance of IAT scores [*F*_(7,531)_ = 20.012; *R*^2^ = 0.21; *AdjustedR*^2^ = 0.20; *p* < 0.001]. As shown in Table 3, IAT scores were significantly positively predicted by ASRS scores, HAD-depression symptoms, UPPS-positive urgency, UPPS-lack of perseverance, and ERQ-expressive suppression scores. ERQ-cognitive reappraisal and UPPS-negative urgency scores were negative predictors of IAT scores. Details are presented in Table 3.

**Table 3:** Multiple regression model explaining IAT score with stepwise method.

	**B**	**Err-type**	**β**	***t*_(530)_**	***p***
OrdOrig.	5.24	0.25		20.863	<0.001^*^
Adult ADHD symptoms	0.151	0.03	0.24	5.342	<0.001^*^
Depression symptoms	0.05	0.02	0.15	3.460	<0.001^*^
Cognitive reappraisal	−0.02	0.01	−0.13	−3.364	<0.001^*^
Positive urgency	0.06	0.01	0.16	3.879	<0.001^*^
Expressive suppression	0.02	0.01	0.12	2.952	0.003^*^
Negative urgency	−0.03	0.01	−0.09	−2.139	0.033^*^
Lack of perseverance	0.03	0.02	0.09	2.002	0.046^*^

* *p < 0.05*;

*β, standardized coefficient; B, unstandardized coefficient; IAT, Internet Addiction Test*.

### Mediation Analysis

The total effect of ASRS on IAT was 0.21 [model: *R*^2^ = 0.11; *F*_(1,531)_ = 62.81; *p* < 0.001]. Table 4 demonstrates the mediating role of UPPS-P, ERQ, and HADS scores on the relationship between ASRS and IAT.

**Table 4:** Mediation models of the association between adult ADHD symptoms and PIU.

**Model**	**Mediators**		**a^**1**^**	**b^**1**^**	**Indirect effect, a × b** **(95% CI)^**1**^**
1	UPPS-NU	Negative urgency	0.05^***^	−0.14	−0.007 (−0.017, 0.001)
	UPPS-LPr	Lack of premeditation	0.08^***^	−0.07	−0.005 (−0.021, 0.010)
	UPPS-LPe	Lack of perseverance	0.14^***^	0.30^**^	0.041 (0.015, 0.069)
	UPPS-PU	Positive urgency	0.03^*^	0.39^***^	0.011 (0.00, 0.025)
	UPPS-SS	Sensation seeking	0.02	−0.13	−0.003 (−0.009, 0.002)
2	ERQ-CR	Cognitive reappraisal	0.02	−0.22^***^	−0.004 (−0.015, 0.008)
	ERQ-ES	Expressive suppression	0.02	0.15^**^	0.003 (−0.004, 0.012)
3	HADS-A	Anxiety symptoms	0.12^***^	0.12^*^	0.015 (0.000, 0.032)
	HADS-D	Depression symptoms	0.17^***^	0.21^***^	0.036 (0.017, 0.056)

1 *Unstandardized coefficients*.

*Bias-corrected bootstrap results for the indirect effect, number of resamples 5,000; a: “path a” effect; b: “path b” effect*;

* *p ≤ 0.05*,

** *p ≤ 0.01*,

*** *p < 0.001*.

**Figure 1:**
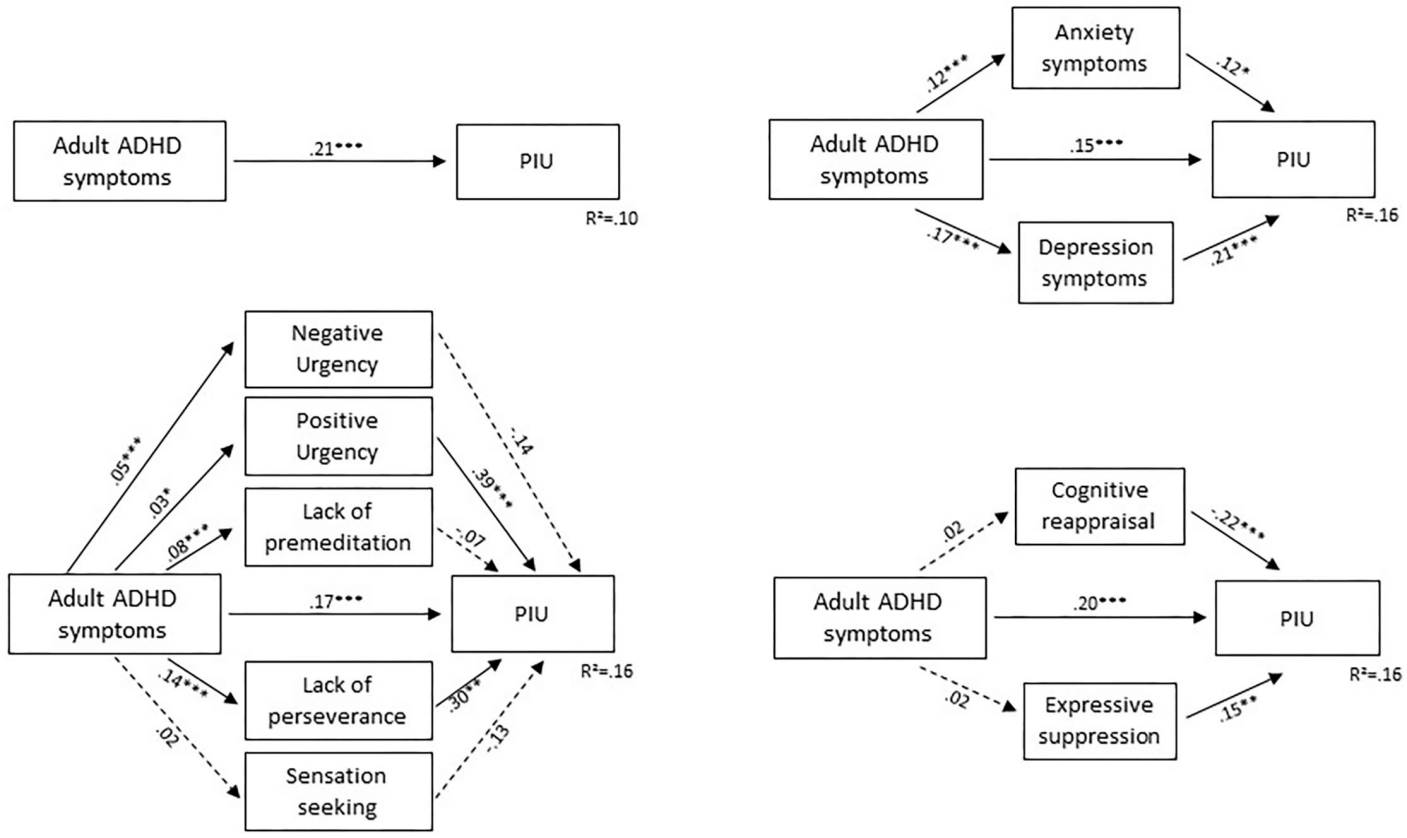
Mediation models of the association between adult ADHD symptoms and PIU. Unstandardized coefficient; *p < 0.05, **p < 0.1, ***p < 0.001; PIU, problematic internet use assessed by the Internet Addiction Test; Non-continuous arrow, non-significant effect; continuous arrow, significant effect.

ASRS and UPPS-P sub-scores significantly predicted IAT scores [*F*_(8,523)_ = 12.19, *p* < 0.001; *R*^2^ = 0.16]. The direct effect of ASRS on IAT (c'-path) was significant (0.17, *p* < 0.001). Therefore, the results suggested that UPPS-positive urgency and UPPS-lack of perseverance scores were partial mediators of the association between ASRS and IAT [indirect effect of positive urgency: 0.011, 95% CI (0.000, 0.025); indirect effect of lack of perseverance: 0.041, 95% CI (0.015, 0.069)]. Details are presented in Table 4 and Figure 1.

ASRS and ERQ sub-scores significantly predicted IAT scores [*F*_(5,526)_ = 19.32, *p* < 0.001; *R*^2^ = 0.16]. The direct effect of ASRS on IAT (c'-path) was significant (0.20, *p* < 0.001). ERQ sub-scores significantly predicted IAT (b-path) but were not predicted by ASRS (a-path). Therefore, emotion regulation sub-scores did not mediate the association between ASRS and IAT. Details are presented in Table 4 and Figure 1.

ASRS and HADS sub-scores significantly predicted IAT scores [*F*_(5,526)_ = 19.32, *p* < 0.001; *R*^2^ = 0.16]. The direct effect of ASRS on IAT (c'-path) was significant (0.15, *p* < 0.001). HADS sub-scores significantly predicted IAT (b-path) and were predicted by ASRS (a-path). Therefore, the results suggested that HADS-anxiety and HADS-depression scores were partial mediators of the association between ASRS and IAT [indirect effect of anxiety: 0.015, 95% CI (0.000, 0.032); indirect effect of depression: 0.036, 95% CI (0.012, 0.056)]. Details are presented in Table 4 and Figure 1.

## Discussion

The purpose of this study was, firstly, to investigate the risk of adult ADHD symptoms in individuals with PIU. Secondly, we investigated how individuals with PIU differed from those without PIU in terms of several psychological factors such as anxiety, depression, impulsivity, emotion regulation and adult ADHD symptoms. Additionally, we investigated the predictive role of these factors (especially adult ADHD symptoms) in PIU severity. Finally, our study aimed to examine the possible mediating role of anxiety, depression, impulsivity and emotion regulation on the relationship between adult ADHD symptoms and PIU. The results of this study showed that the proportion of respondent who screened positive for adult ADHD symptoms was higher for individuals with PIU. Moreover, they had higher scores for anxiety and depressive symptoms, impulsivity (especially lack of perseverance, and premeditation), and expressive suppression and lower scores for cognitive reappraisal, than those without PIU. PIU severity was positively predicted by adult ADHD symptoms, depressive symptoms, positive urgency, lack of perseverance, and expressive suppression, and was negatively predicted by cognitive reappraisal and negative urgency. Finally, anxiety and depressive symptoms, positive urgency, and lack of perseverance were partial mediating factors of the association between adult ADHD symptoms and PIU severity.

Firstly, the results of this research confirm the significantly higher proportion of individuals with PIU who screened positive for adult ADHD symptoms, than those without PIU. These findings are in line with those of previous studies investigating ADHD-PIU comorbidity (8, 43–45). Therefore, it appears that individuals with PIU compared to those without are more likely to present with comorbid adult ADHD symptoms. Moreover, the mean age of our sample was young. And, the risk of engagement in problematic behaviors is higher in adolescents and young adults (46), especially if ADHD symptoms co-occurred (47). These results question the causal relationship between PIU and adult ADHD symptoms. Are PIU and adult ADHD independently caused by similar risk factors or does one cause the other?

Comparative analyses highlighted the clinical features of individuals with PIU, thus providing a better understanding of their function in terms of impulsivity, emotion regulation, and anxiety-depressive symptoms. Based on the results of this study, these individuals showed greater impulsivity, with significantly higher scores on lack of premeditation, and lack of perseverance, than those without PIU. Taken together, individuals with PIU therefore generally showed more marked impulsivity than those without PIU. These results are in line with a study conducted by de Vries et al. (44), which found that individuals with PIU had higher scores on the Barratt Impulsiveness Scale than those without PIU. In addition, the analyses of emotion regulation in this study indicated that expressive suppression was significantly higher, whereas cognitive reappraisal was significantly lower in people with PIU than individuals without PIU. The results of the linear multiple regression analysis showed that certain factors may explain the severity of PIU. Indeed, adult ADHD symptoms, depression symptoms, positive urgency, and expressive suppression were all predictors of the severity of PIU. Additionally, the results suggested that cognitive reappraisal can protect against the development of PIU, and negative urgency negatively predicts the disorder's severity. According to Gross and John (35), expressive suppression is associated with rumination about events that make the individual feel bad, high levels of negative emotions and depressive symptoms. These results highlight the greater vulnerability of individuals with PIU, as they tend to use ineffective emotion regulation strategies and to be more impulsive than those without PIU. The hypothesis that individuals with PIU have greater difficulty regulating their emotions has also been suggested by Koronczai et al. (48) and Przepiorka et al. (49). These results are in line with previous publication which highlighted the predictive role of emotion dysregulation on addictive behaviors (50). Engagement in addictive behaviors may be a way to avoid or regulate negative emotions, and “prolong or extend positive emotional states, if they demonstrate poor regulation over their emotions or lack alternative ways of responding” Estévez et al. (50). Depression may be a mediator between emotional stability and PIU (48), thus may explaining the significantly higher anxiety and depression scores of our participants with PIU.

It is highly likely that individuals with adult ADHD symptoms have important emotional dysregulation difficulties (18) and are at significant risk of using inappropriate strategies to cope with life events. Emotional impulsivity (18), anxiety and mood disorders (51, 52) are frequently observed in individuals with ADHD. “One important consideration is the possibility of depressive symptoms manifesting as a result of coping with lower hedonic tone in ADHD rather than being representative of a depressive disorder separate from ADHD” (53). Previous publications have suggested that impulsivity in ADHD may stimulate addictive behavior (54). Based on research with gambling disorders, three profiles of problem behaviors can be identified: behaviorally conditioned, emotional vulnerability, and anti-social impulsivity (7). The emotionally vulnerable and the anti-social impulsivity profiles may partly apply to individuals with adult ADHD symptoms, thus explaining their high risk of problematic behavior, especially in terms of PIU. Di Nicola et al. (52) suggest individuals with mood disorders and comorbid ADHD has a higher risk of suicide attempts, and lifetime substance use disorder, than individuals without comorbid ADHD. Additionally, individuals with problematic behaviors who report ADHD symptoms have higher levels of impulsivity, anxiety disorders, negative emotionality, and lower positive emotionality than individuals without ADHD (55). Individuals with PIU are at higher risk of having comorbid adult ADHD symptoms than those without PIU, which may be expressed through more marked impulsivity and emotion regulation difficulties (18). These results suggest either these two disorders have similar dysfunction-related underpinnings (features such as poor emotion regulation strategies, impulsivity and negative affectivity), one disorder represents an unsuccessful attempt to regulate the other or that the causality is bi-directional.

Finally, the mediation analyses provided a better understanding of the possible mediating effects of variables including impulsivity, emotion regulation, and anxiety-depressive symptoms on the co-occurrence of adult ADHD symptoms and PIU. The results demonstrated a mediating role of impulsivity through two of its sub-dimensions (positive urgency and lack of perseverance), as well as of anxiety and depressive symptoms. Conversely, emotion regulation, through expressive suppression and cognitive reappraisal, did not have a mediating effect on the comorbidity of these two disorders. It would be interesting to look at that issue in more depth. Further studies should use the Difficulty in Emotion Regulation Scale [DERS; (56)], which identifies 6 sub-dimensions of emotion dysregulation: non-acceptance, goals, impulse, strategies, awareness, and clarity. Indeed, the use of this multidimensional model and its corresponding questionnaire could reveal difficulties in emotion regulation in individuals with comorbid adult ADHD-PIU, which could then provide a useful basis for therapeutic interventions. Kalbag and Levin (57) suggested cognitive-behavioral therapy to manage both ADHD and problematic behavior such as substance abuse. They reported the importance of managing poor coping skills and strategies, control of emotional reactions, feelings of being overwhelmed by negative life events, and negative emotions, which in turn will reduce substance reliance. In line with the literature, we hypothesize impulsivity, mood, and anxiety disorders may further increase the risk of problematic behaviors such as PIU, even more for individuals who screen positive for adult ADHD symptomatology. The current results agree the hypothesis that the use of the Internet may be a way of coping with the difficulties arising from having comorbid adult ADHD symptoms. Therapeutic and preventive interventions targeting emotional impulsivity and anxiety-depressive symptoms may be contemplated to prevent and manage comorbid PIU and adult ADHD symptoms.

This study has some limitations. The cross-sectional design and the lack of investigation of the comorbid addictive behaviors prevent any conclusions about causality. Future studies should opt for a longitudinal design and plan investigation of comorbid addictive disorders to ensure that the differences between groups are associated with PIU, not other addictive behavior. Future investigations should also screen for comorbid psychiatric disorders. The study was carried out with a sample of non-clinical adults, and it would be beneficial to conduct further investigations with a clinical population of adults with diagnosed ADHD or PIU, who have more marked psychopathological features. Additionally, all data was collected online, and the participants were recruited on the social media. We did not have access to the number and the characteristics of the total potential participants, for which the questionnaire was visible. This questions the representativeness of the sample. We also used self-administered questionnaires. For example, the ASRS has limitations in terms of screen for adult ADHD symptoms. Using a semi-structured diagnostic interview for adult ADHD, such as the Diagnostisch Interview Voor ADHD bij volwassenen (DIVA), which assesses the occurrence of DSM-5 ADHD criteria (inattention, hyperactivity, and impulsivity) across both childhood and adulthood, may strengthen the results presented in the current paper. We also did not consider the effect of different Internet activities on PIU. Therefore, PIU may vary and possibly involve different psychopathological mechanisms depending on the type of Internet activity. For example, previous investigations have found that PIU in adolescents with ADHD is specifically associated with online gaming, emailing, and social networking (58). Their results emphasize that PIU is associated with specific online activities, which warrants further in-depth investigation.

In conclusion, the results of this research confirmed the high levels of comorbidity between adult ADHD symptoms and PIU in the general population. The current study also highlighted the need for further investigation of PIU in individuals with adult ADHD symptoms and of adult ADHD symptoms in individuals with PIU in order to reduce the co-occurrence of these two disorders, which may lead to negative outcomes. Individuals with PIU presented characteristics similar to those observed in individuals with adult ADHD symptoms, including in terms of high levels of impulsivity and its sub-dimensions, difficulties with emotion regulation, and anxiety and depression. The results of the impulsivity sub-dimensions and anxiety-depressive symptoms were in line with a mediating effect in the relationship between the two disorders. Therefore, future studies should investigate clinical interventions targeting both adult ADHD and PIU by focusing on impulsivity, emotion dysregulation, and comorbid psychiatric disorders such as anxiety and depression. This study conducted in non-clinical population identified psychopathological risk factors of PIU and enable the identification of vulnerable individuals who prevention interventions may target. Adolescents and young adults are especially at risk for problematic behaviors, mood and anxiety disorders. Therefore, further investigations in this specific population are needed.

## Data Availability Statement

The raw data supporting the conclusions of this article will be made available by the authors, without undue reservation.

## Ethics Statement

The studies involving human participants were reviewed and approved by IRB 2019-03-01. The patients/participants provided their written informed consent to participate in this study.

## Author Contributions

SB: data collection. SB, AR, and SE: writing—original draft preparation and writing—review and editing. IV, PB, and SB: study design, concept, and supervision. All authors have read and approved the published version of the manuscript.

## Funding

SE's PhD work was funded by a Presidential University Grant (University of Tours).

## Conflict of Interest

The authors declare that the research was conducted in the absence of any commercial or financial relationships that could be construed as a potential conflict of interest. The Handling Editor FV declared a shared affiliation, though no other collaboration, with the author IV at the time of the review.

## Publisher's Note

All claims expressed in this article are solely those of the authors and do not necessarily represent those of their affiliated organizations, or those of the publisher, the editors and the reviewers. Any product that may be evaluated in this article, or claim that may be made by its manufacturer, is not guaranteed or endorsed by the publisher.
